# Removal of luminal content protects the small intestine during hemorrhagic shock but is not sufficient to prevent lung injury

**DOI:** 10.1002/phy2.109

**Published:** 2013-10-20

**Authors:** Angelina E Altshuler, Michael D Richter, Augusta E Modestino, Alexander H Penn, Michael J Heller, Geert W Schmid-Schönbein

**Affiliations:** 1 Department of Bioengineering, The Institute of Engineering in Medicine, University of California San DiegoLa Jolla, California, 92093-0412; 2 Department of Nanoengineering, The Institute of Engineering in Medicine, University of California San DiegoLa Jolla, California, 92093-0412

**Keywords:** Hemorrhagic shock, lung injury, lymph, protease activity, small intestine

## Abstract

The small intestine plays a key role in the pathogenesis of multiple organ failure following circulatory shock. Current results show that reduced perfusion of the small intestine compromises the mucosal epithelial barrier, and the intestinal contents (including pancreatic digestive enzymes and partially digested food) can enter the intestinal wall and transport through the circulation or mesenteric lymph to other organs such as the lung. The extent to which the luminal contents of the small intestine mediate tissue damage in the intestine and lung is poorly understood in shock. Therefore, rats were assigned to three groups: No-hemorrhagic shock (HS) control and HS with or without a flushed intestine. HS was induced by reducing the mean arterial pressure (30 mmHg; 90 min) followed by return of shed blood and observation (3 h). The small intestine and lung were analyzed for hemorrhage, neutrophil accumulation, and cellular membrane protein degradation. After HS, animals with luminal contents had increased neutrophil accumulation, bleeding, and destruction of E-cadherin in the intestine. Serine protease activity was elevated in mesenteric lymph fluid collected from a separate group of animals subjected to intestinal ischemia/reperfusion. Serine protease activity was elevated in the plasma after HS but was detected in lungs only in animals with nonflushed lumens. Despite removal of the luminal contents, lung injury occurred in both groups as determined by elevated neutrophil accumulation, permeability, and lung protein destruction. In conclusion, luminal contents significantly increase intestinal damage during experimental HS, suggesting transport of luminal contents across the intestinal wall should be minimized.

## Introduction

The digestive track has long been associated with the progression of shock and multiple organ dysfunction syndrome (MODS) in intensive care units (Lillehei 1957; Robinson et al. 1966), and the small intestine in particular is a major contributor to the dysfunction (Lillehei 1957; Poggetti et al. 1992; Magnotti et al. 1998). Hypovolemia from hemorrhage results in intestinal ischemia and subsequent apoptosis of epithelial cells (Lu et al. 2008). This results in epithelial shedding and loss of the attached mucin (Ikeda et al. 1998; Grossmann et al. 2002; Sheng et al. 2011; Chang et al. 2012a,b). Together the mucin and epithelial tight junctions form the mucosal barrier that is responsible for keeping the intestinal contents, including pancreatic digestive enzymes and digested food particles, compartmentalized in the intestine's lumen. Failure of the barrier as a result of ischemia allows the contents of the intestine to penetrate into the wall of the intestine and contribute to further intestinal damage (Chang et al. 2012a,b).

The presence of food in the intestine is a major factor that influences the outcome of shock conditions (Bounous 1967; Bounous et al. 1967). The luminal contents in the small intestine consist of digestive enzymes, food particles, bile, and some bacteria (far less than in the large intestine). They may be nonuniformly distributed along its length depending on the time of food ingestion and vary between trauma patients. Luminal contents may serve as proinflammatory mediators after the onset of intestinal ischemia contributing to the MODS that occurs during shock if they escape the intestine via the mesenteric lymph, circulation, or via transmural permeation into the peritoneal space where they may initiate inflammation in peripheral organs, for example, the lung (Ishimaru et al. 2004; Deitch 2010; Altshuler et al. 2012).

However, the benefits from the nutrient component of the luminal contents, for example, sugars and amino acids, help to maintain the mucosal epithelial barrier during intestinal ischemia (Chiu et al. 1970b; McArdle et al. 1972; Robinson and Mirkovitch 1977; Flynn et al. 1992), and thus may be of advantage to the intestine during shock. The main drawback of digesting food is that it is dissolved in a mixture of powerful degrading enzymes in the lumen of the intestine that may cross the mucosal epithelial barrier during the ischemic state (Chang et al. 2012b) when the epithelial layer becomes permeable (Ikeda et al. 1998; Grossmann et al. 2002). Inhibition of pancreatic enzymes in the lumen of the intestine reduces intestinal microhemorrhages, decreases distant organ injury, and improves survival after experimental shock (Mitsuoka and Schmid-Schönbein 2000; Mitsuoka et al. 2000; Deitch et al. 2003; Doucet et al. 2004; Shi et al. 2004; DeLano et al. 2013), suggesting a prime role for these enzymes in shock pathogenesis. However, the inhibitors may not only act on the enzymes within the intestinal lumen. If the inhibitors themselves cross the mucosal barrier, they may also affect enzymes in the intestinal wall, vasculature, and/or other organs. An alternative method for determining the contribution of the luminal content to hemorrhagic shock (HS) is needed, for example, flushing of the entire luminal content in a segment of the intestine that has previously been the target of intraluminal protease inhibitor injections prior to hypotension.

Therefore the aim of this study was to explore the contribution of small intestine luminal contents to intestinal and peripheral organ injury following HS. We hypothesized that luminal contents including digestive enzymes enter into the interstitial mucosal wall and cause microhemorrhages in peripheral organs by accumulation of proteases and activated neutrophils. The results suggest that in the absence of luminal contents in the small intestine, intestinal injury and protease accumulation in the lung is abrogated, but lung injury at 3-h postreperfusion is not reduced.

## Methods

### Animals

The animal protocol was reviewed and approved by the University of California, San Diego Institutional Animal Care and Use Committee (Protocol Number S01113). Male Sprague Dawley (for HS model; mean body weight 240–300 g, *N* = 18, Harlan, Indianapolis, IN) or male Wistar rats (for lymph collection model; 250–350 g, *N* = 10, Harlan) were allowed food and water ad libitum prior to surgery. Rats were administered general anesthesia (xylazine, 4 mg/kg; ketamine 75 mg/kg i.m.) and euthanized by infusion of B-Euthanasia i.v. (120 mg/kg) at the termination of experiments. Following general anesthesia, the femoral artery and vein were cannulated. Systolic, diastolic, heart rate, and mean arterial pressure (MAP) were recorded throughout the procedure using LabChart (AD Instruments, Dunedin, New Zealand).

### HS procedure

#### HS with removal of luminal contents

Animals were grouped into no-HS (No-HS), HS with intestinal luminal contents *flushed* (HS-F), and HS *without intestinal flush* (HS-NF). No-HS animals were cannulated and then immediately sacrificed for tissue collection. After femoral vessel cannulation, the HS-F and HS-NF were subject to laparotomy, and the intestine was exposed. The proximal jejunum (∼5 cm distal from the ligament of Treitz) was carefully cut between intestinal blood vessels and each end was cannulated with Female Luer to Barb tube connectors and secured with 4-0 suture. The connector cannulated to the duodenum was sealed with clay to prevent entry of new pancreatic proteases into the jejunum and ileum or peritoneal space after the intestine was flushed. Next, the distal ileum located three centimeters from the cecum was cut in order to insert another clay sealed connector into the intestine on the distal side (closest to the cecum) and secured with suture. The proximal end was not cannulated and sealed until after the luminal contents were flushed.

To remove the luminal contents, a syringe filled with 40 mL of saline at 37°C was connected to the adaptor attached to the proximal jejunum, and the luminal contents were flushed distally with saline using pulsatile pressure. The contents exiting the intestine through the distal ileum were collected and discarded. Using this technique, all visible luminal content was removed from the intestine, and the last ∼15 mL of saline exiting the intestine were clear of color and absent of solid food residues. Following the intestinal flush, the final connector in the distal ileum was inserted and secured with suture. All connectors were sealed with clay. The exterior of the intestine was rinsed with warm saline (37°C) and placed into the peritoneal cavity. HS-NF animals received the same manipulation and placement of connectors in the intestine, but the luminal contents were not removed. Gross morphology images of pre-HS intestines were recorded immediately prior to replacement in the peritoneal cavity.

Following the intestinal preparations, the animals were heparinized to minimize clotting in catheters and in shed blood samples (1 U/mL of calculated blood volume i.v.; assuming 6 mL blood volume per 100 g total body weight) before onset of hemorrhage. MAP was reduced to 30 mmHg by withdrawal of blood through the venous catheter (0.4 mL/min) with a 5 mL syringe. The initial 1 mL of blood drawn was collected, centrifuged (1000*g*, 5 min), and the plasma was immediately frozen at −80°C for further analysis. The arterial pressure was monitored and adjusted by withdrawal/return of blood over the 90-min ischemic period. At the end of ischemia, the shed blood was returned to the animals (0.5 mL/min) plus 1 mL of saline (to replace the 1 mL of blood collected before shock). The animals remained anesthetized and were observed in the reperfusion phase for 3 h after initiation of the return of shed blood.

Gross morphology images of the intestine were captured at the end of the reperfusion period. Post-HS blood was collected through the arterial catheter, spun (1000*g*, 5 min), and the plasma was immediately frozen at −80°C. The animals were then euthanized and lung and jejunum specimen were snap frozen for homogenization or embedded in optimum cutting temperature (O.C.T.) (Saukura, Leiden, the Netherlands) for histological sectioning. Jejunum was chosen for analysis, as we typically see more intestinal hemorrhage there than in the ileum in models of HS.

#### Lymph collection during intestinal ischemia

As a positive control to demonstrate that active pancreatic proteases transport through the mesenteric lymph following shock, we collected mesenteric lymph and measured trypsin and chymotrypsin levels and activity over the course of intestinal ischemia. After a 3-cm midline laparotomy, the intestine was relocated from the abdomen and placed on a 1 cm platform to the left of the animal and covered with saline-soaked gauze and saran wrap to retain moisture, exposing the base of the mesentery and the superior mesenteric lymph vessel. A 1-cm bridge was placed under the animal to further expose the lymph vessel, which was then cleared of fat and surface fascia using blunt dissection. Silastic tubing (0.64 mm internal diameter) prefilled with heparin (10 mg/mL) to prevent clotting was drawn through the right abdominal wall using a suture needle and looped under the vena cava using curved tweezers. The superior mesenteric lymph vessel was cannulated with the tubing and held in place with VetBond tissue glue (3M, St. Paul, MN).

Once lymph fluid, visible as a white cloudy fluid, reached the other end of the cannula, this catheter end was placed inside a 2 mL tube preloaded with 5 μL of heparin. Fluid was collected for 1 h under the same conditions for every animal. Intestinal ischemia was induced by splanchnic arterial occlusion (SAO; *N* = 5). Two microclamps were used to occlude the superior mesenteric artery and the celiac artery for the duration of the second hour, while animals in the Sham group (*N* = 5) remained perfused. After the second hour, the clamps were removed from the SAO animals to begin reperfusion. Lymph fluid was collected continuously for another 3 h and aliquoted every hour. At the end of each hour, samples were centrifuged (1600*g*, 4°C, 20 min) to remove cellular debris and excess fat, and the supernatant was stored at −80°C. After the 5 h of lymph fluid collection, animals were euthanized (120 mg/kg sodium pentobarbital i.v.).

### Bronchoalveolar lavage fluid

After HS, bronchoalveolar lavage fluid (BALF) was collected by cannulating the left main bronchus leading to the left lung with an 18 gauge blunt needle. The lung was photographed to assess gross morphological damage. The images were blindly assessed for macroscopic lung damage. Scoring was as follows: 0, no visible lung damage and pink in color; 1, mostly pink in color with some dark red lesions starting to form around the central part of the lung near the trachea; 2, some pink color with more lesions forming and spreading throughout the lung; 3, dark red lesions through the majority of the lung surface; 4, complete lung damage in all parts of the organ surface.

To collect BALF fluid, saline (∼2.0 mL/lavage) was injected and recovered three times before centrifugation (1000*g*, 5 min). Following the third wash, the lung was injected with O.C.T., embedded, and frozen. The total volume of recovered BALF fluid was measured and the fraction of saline recovered versus the amount injected was used to normalize measurements.

### Tissue homogenization and analysis

In every homogenization procedure (excepting those for immunoblotting, see below), 1 mL of 0.5% hexadecyltrimethylammonium bromide (HTAB) in phosphate buffered saline (PBS) (pH 6.0) was added per 0.1 g tissue. Tissues were then mechanically homogenized using a handheld homogenizer for 30 sec before centrifuging (18,000*g*, 20 min). Supernatants were stored at −80°C until processing.

All microplate assays were performed in duplicate in 96 well black-sided flat bottom polystyrene plates (Corning, New York, NY) and measured in a microplate reader (FilterMax F-5 Multi-mode; Molecular Devices, Sunnyvale, CA).

### Intestinal hemorrhage

Erythrocyte infiltration, that is, hemorrhage, was estimated in homogenates of jejunum, including any luminal content present in the segment, by reading absorbance at 405 nm, an absorbance peak for hemoglobin, in duplicate on a plate reader. Intestinal bleeding was also assessed macroscopically by comparing photographs of the intestine before and after HS.

### Enzyme and protease activity measurements

#### Casein activity in the intestine

Protease activity was determined by digestion of the globular protein casein hybridized to Texas-red such that fluorescence intensity increases after cleavage (Enzchek protease assay kit; Life Technologies, Carlsbad, CA). Jejunal homogenates (10 μL) including any endogenous luminal contents present were mixed with casein substrate (90 μL) and loaded in duplicate. Plates were incubated in a prewarmed microplate reader and measurements were made every 5 min. The initial slope (i.e., change in caseinolytic activity per minute) was computed for each sample.

#### Myeloperoxidase activity assay

In order to assess intestine and lung neutrophil infiltration, myeloperoxidase (MPO) activity was determined in the intestine and lung homogenates. Protein content was measured (BCA kit; ThermoScientific, Waltham, MA). Forty microliter (2 mg/mL) intestine homogenate or 20 μL (1 mg/mL) lung homogenate were added to 180 μL of PBS (pH 6.0) mixed with 0.167 mg/mL o-dianisidine dihydrochloride (Sigma-Aldrich, St. Louis, MO) and 0.001% or 0.0005% H_2_O_2_ (w/v) for intestine and lung, respectively.

To determine neutrophil MPO release into the BALF, 20 μL was loaded into each well before 180 μL of PBS (pH 6.0) mixed with 0.167 mg/mL o-dianisidine dihydrochloride (Sigma-Aldrich) containing 0.001% H_2_O_2_. BALF protein concentration was also determined. All BALF calculations were normalized to the percent fluid recovered from three lavage collections.

Absorbance was measured kinetically at 450 nm every 5 min for 1 h at 37°C. As negative controls, 180 μL of PBS (pH 6.0) was added to 20 μL of sample, and the absorbance values of these samples were subtracted from the measurements with the substrate. The change in absorbance was linear within this period, and MPO activity is presented as the change in absorbance per minute per milligram of protein.

### Charge separating substances

To measure the activity of specific pancreatic enzymes in lymph, we used a new electrophoretic method (Lefkowitz et al. 2010). The method is superior to colorimetric assays as the lymph often has an opacity (due to light diffraction) that interferes with absorbance measurements. These substrates are small peptide sequences that carry opposing charges at each end. When they are cleaved, the end with an attached fluorophore has a net positive charge, making it possible to isolate the cleaved segment with electrophoresis. A trypsin-specific substrate (acetyl-N-DGDAGRAGAGK-NH_2_) synthesized by Aapptec (Louisville, KY) was labeled on the lysine residue's ε amine group with Bodipy FL-SE (Invitrogen, Carlsbad, CA). A similar chymotrypsin-specific peptide (acetyl-N-DGDAGYAGLRGAG-diamino ethyl- Bodipy, FL) was also synthesized. Note that while the cleavage sites of these substrates are designed for trypsin and chymotrypsin, it is possible for other proteolytic enzymes with similar specificities to cleave them at a slower rate. Solutions of each substrate (0.5 mg/mL) were combined with 3.5 μL of lymph fluid in individual reaction tubes, and allowed to react for 1 h. Aliquots of each sample were electrophoresed at 500 volts (10 min), in a precast 20% polyacrylamide gel (Life Technologies). Fluorescent bands were quantified using a Storm 840 scanner (Molecular Dynamics, Sunnyvale, CA) configured to ImageQuant v5.2 software (General Electric, Fairfield, CT) using the following settings: fluorescence mode, high sensitivity, 100 mm pixel size, 1000 V photomultiplier tube with a 450 nm excitation filter and a 520 nm long pass emission filter. Digital images were integrated over each sample band followed by background subtraction (negative control, substrate + HCl) to obtain the final fluorescence values (ImageJ; http://rsb.info.nih.gov/ij/).

### Gelatin gel zymography

When present, the luminal contents from the jejunal tissue were discarded by gentle compression of the intestine before homogenization to prevent excess digestion of the gelatin substrate in the gels by luminal proteases. Tissues from shocked animals (flushed and nonflushed) as well as controls were rinsed and blotted dry before weighing. Protein concentration was determined using the BCA kit before mixing each sample in a 1:1 ratio with loading dye (containing sodiumdodecyl sulfate [SDS], but no reducing agent). Two microgram protein/lane of intestine or 20 μg protein/lane of lung homogenates were loaded. Plasma (0.5 μL) was mixed with 2 μL loading dye and loaded into each lane.

Samples were separated by gel electrophoresis in 11% sodiumdodecyl sulfate polyacrylamide gel electrophoresis (SDS-PAGE) gelatin impregnated gels. Upon separation, gel proteins were renatured in 2.5% Triton-X 100 in water (4×, 15 min) before incubation at 37°C overnight in developing buffer (0.05 mol/L Tris base, 0.2 mol/L NaCl, 4 μmol/L ZnCl_2_, 5 mmol/L CaCl_2_**·**2H_2_O). Gels were then fixed and stained (50% methanol, 10% acetic acid, 40% water, and 0.25% Coomassie blue solution) for 3 h, destained in water to achieve appropriate contrast before digital analysis (ImageJ).

To confirm that the ∼20-kDa bands were serine proteases, gels were renatured and developed in the aforementioned buffers with the addition of either 75 μmol/L serine protease inhibitor ANGD (nafamostat mesilate) or 100 μmol/L trypsin inhibitor tosyl-lysine chloromethyl ketone (TLCK) and compared to duplicate gels that were subject to the standard renaturation procedure.

### Immunoblotting

Before homogenizing intestinal samples for immunoblotting, the luminal content was gently pressed out of the intestine for the No-HS and HS-NF samples. The intestines were rinsed in saline and blotted dry before weight determination. Lysis buffer (ThermoScientific) was mixed with a 1:100 dilution of 0.5 mol/L ethylenediaminetetraacetic acid (ThermoScientific) and a 1:50 dilution of HALT protease inhibitor cocktail. Lungs were homogenized in lysis buffer containing HALT protease inhibitor (1:100). A ratio of 1 mL of lysis buffer per 0.1 g of tissue was used for both organs.

For organ homogenates, protein concentration was calculated (BCA kit) so that 40 μg protein was loaded per well. Homogenates were mixed 1:1 with sample loading buffer (Bio-Rad, Hercules, CA) containing β-mercaptoethanol (0.05% by volume). Plasma was mixed in a 1:2 ratio of plasma:sample loading buffer containing β-mercaptoethanol (0.05% w/v), and 3 μL of this solution was loaded per well. Lymph fluid was mixed in a 1:1 ratio of lymph fluid:sample loading buffer containing β-mercaptoethanol (0.05% w/v), and 5 μL of this solution was loaded per well. Both tissue homogenates and plasma samples were denatured by boiling for 10 min prior to loading. Proteins were separated on SDS-PAGE gels (8% or 12% resolving, 4% stacking). Following separation, proteins were transferred onto a nitrocellulose membrane (Bio-Rad) and blocked for 1 h in 5% bovine serum albumin in tris-buffered saline with 0.5% Tween-20 (TBS-T). Membranes were cut longitudinally near the anticipated molecular weight of each protein and incubated overnight with the designated primary antibody (β-actin, Santa Cruz Biotechnology (Santa Cruz, CA), sc-8432, 1:1000; chymotrypsin, Abcam (Cambridge, MA) ab-35694, 1:500; E-cadherin, Invitrogen 33-4000, 1:1000; matrix metalloproteinase-9 (MMP-9), Abcam ab-76003, 1:1000; mucin 13, Santa Cruz Biotechnology sc-66973, 1:1000; occludin, Invitrogen 33-1500, 1:1000; Trypsin, Santa Cruz Biotechnology sc-137077, 1:1000; VE-cadherin, Santa Cruz Biotechnology sc-6458, 1:1000; VEGFR-2, Santa Cruz Biotechnology sc-48161, 1:750).

Following incubation, the primary antibody was rinsed (TBS-T, 3×, 10 min). Secondary antibodies against mouse, rabbit, or goat were diluted 1:10,000 in TBS-T and incubated with the membranes for 1 h. The membranes were washed (TBS-T, 3×, 10 min) and developed using enhanced chemiluminescence substrate (ThermoScientific).

Membranes were scanned and bands digitally quantified (ImageJ). Lung and intestine bands were standardized by β-actin and normalized by the No-HS controls to determine fold changes in protein levels. Plasma protein levels were not normalized.

### Histology

All procedures were performed on 8-μm thick frozen sections. Images were acquired using a 20× objective and montaged together.

#### Immunohistochemistry

Mucin distribution was assessed using immunohistochemistry after HS. Sections were allowed to dry for 20 min before fixation in ice-cold acetone at −20°C for 10 min. Following fixation, slides were washed in PBS (2×, 3 min). Endogenous hydrogen peroxidase activity was quenched (95% methanol, 3% hydrogen peroxide for 5 min). Slides were washed again in PBS (2×, 3 min). Individual sections were isolated with a hydrophobic pen (Vector Labs, Burlingame, CA) before blocking with 2.5% normal horse serum (Vector Labs) for 30 min. A primary antibody against mucin 13 (Santa Cruz Biotechnology; sc-66973) was diluted 1:200 in 1.25% normal horse serum and incubated with the sections overnight at 4°C. The primary antibody was washed with PBS (3×, 5 min) and sections were incubated with anti-rabbit secondary reagent (Immpress Kit; Vector Labs) for 30 min. Secondary antibody was removed by washing with PBS (3×, 5 min). Colorimetric labeling was achieved by using DAB (3, 3′-diaminobenzidine) substrate (Vector Labs) for 30 sec followed by rinsing in water. Sections were finally counterstained with Toluidine blue (0.05% in 1% boric acid) for 5 sec. Slides were washed with three changes of deionized (DI) water before dehydrating in an ethanol gradient (70%, 95%, and 100%) and cleaning with xylene. Slides were allowed to dry overnight before mounting with hard set mounting media (Vector Labs).

#### Lung histology

Lung sections were allowed to dry for 20 min before fixation in ice-cold methanol at −20°C. Slides were washed in DI water (Bounous 1967) and incubated with hematoxylin solution (Vector Labs) for 30 sec. They were rinsed thoroughly in DI water and incubated in eosin (Fisher Scientific, Waltham, MA) for 30 sec followed by additional rinses in DI water. The slides were dehydrated in 70%, 95%, and 100% ethanol before cleaning with xylene and were dried overnight before being mounted with hard set mounting media (Vector Labs).

### Statistical analysis

Results are presented as either mean ± standard deviation (SD). Paired *t*-tests were completed for comparisons between groups for MAP during the reperfusion period and between pre- and postshock plasma samples. T-tests were used to compare Sham and SAO lymph fluid protease activities. Mann–Whitney tests were used for nonnormally disturbed data. Analyses of variance (ANOVA) followed by Tukey post hoc analysis was used for normally distributed parametric variables. Statistical analysis was performed with commercial software (OriginLabs, Northampton, MA).

## Results

### Intestinal injury occurs if luminal contents are present

**Figure 1:**
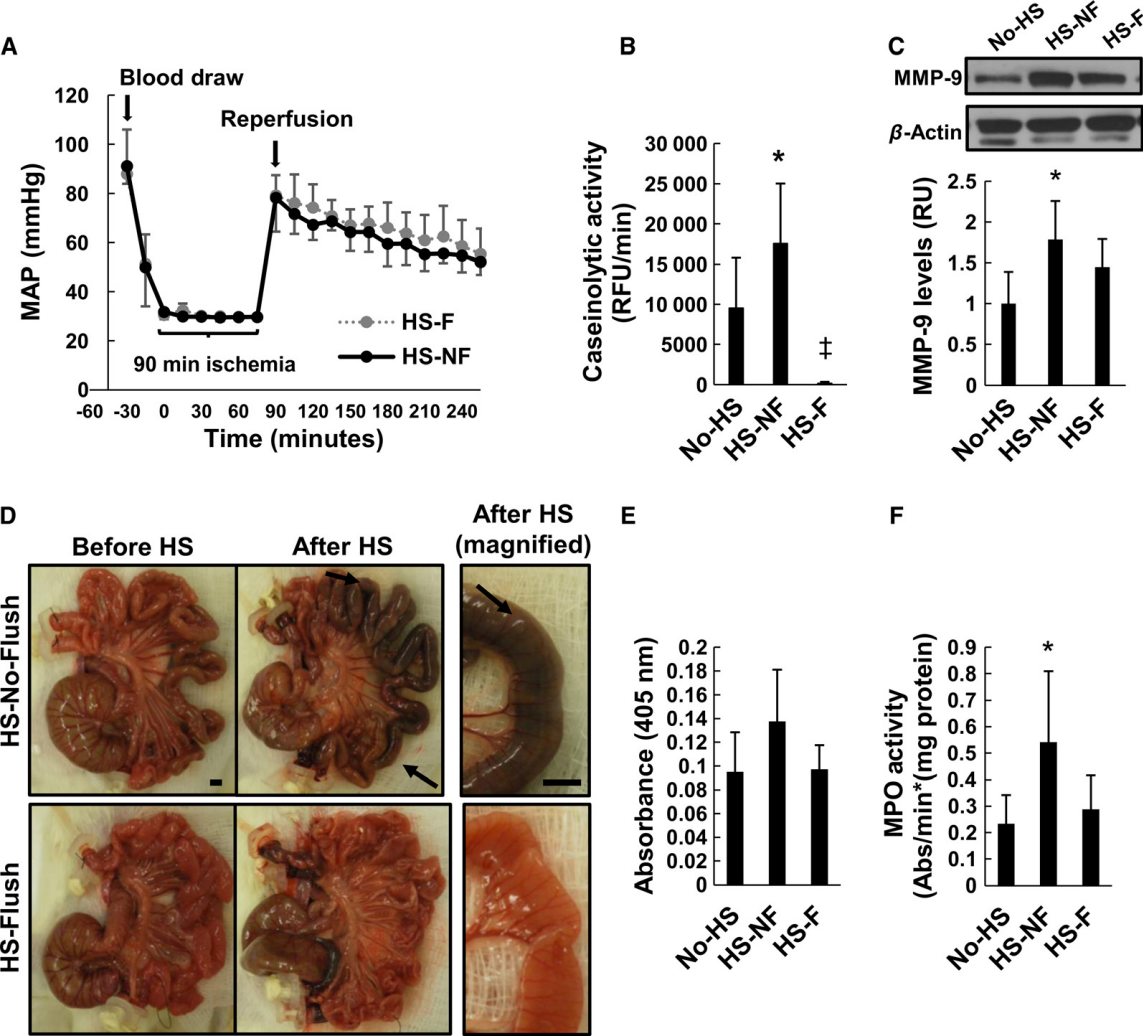
Intestinal injury caused by luminal contents. (A) The MAP (mean ± SD) during hemorrhagic shock (HS) for groups of rats with flushed (HS-F) and nonflushed (HS-NF) intestine. (B) Protease activity in intestinal homogenates as measured by caseinolytic activity. (C) MMP-9 density and quantification in intestinal homogenates. (D) Gross intestinal morphology following HS with either a flushed or nonflushed intestine. Black arrows indicate sites of hemorrhagic lesions. (E) Hemoglobin absorbance measured at 405 nm of intestinal homogenates. (F) MPO activity in intestinal homogenates. N = 6 rats/group. Bar graphs show mean ± SD. *P < 0.05 compared to No-HS and ^‡^compared to HS-NF by ANOVA followed by Tukey post hoc analysis. Scale bar corresponds to 5 mm.

The blood pressure between the HS-NF and HS-F animals did not significantly differ over the observation period of 180 min (Fig. 1A). As expected, given the presence of luminal content and proteases, the intestinal proteolytic activity as determined by casein substrate digestion in the HS-NF animals was significantly higher compared to flushed animals (Fig. 1B). Additionally, the activity increased in the HS-NF group compared to the No-HS group, suggesting local activation and/or influx from the circulation of proteases. MMP-9, detected by immunoblot, was elevated in the HS-NF animals compared to the No-HS animals (Fig. 1C). All animals had intestines without lesions prior to the onset of ischemia/reperfusion injury, but the intestines developed microhemorrhages along the length of the intestine in both the jejunum and ileum regions of the HS-NF group only (Fig. 1D). These lesions appeared more intense at sites where food was present. In contrast, none of the six HS-F animals had macroscopic lesions except near the surgical sites at the proximal jejunum and distal ileum (a feature shared by the HS-F and HS-NF animals). Though the intestinal homogenate absorbance (at 405 nm) as a quantitative marker for hemoglobin escape was not significantly different between the groups (*P* = 0.08 between No-HS and HS-NF; Fig. 1E), the hemoglobin absorbance linearly correlated with the degree of caseinolytic activity in the nonflushed samples (*R*^2^ = 0.88; not shown). MPO activity was also elevated in the HS-NF animals but not the HS-F animals (Fig. 1F), suggesting that the MMP-9 elevation in Figure 1C may be neutrophil derived.

**Figure 2:**
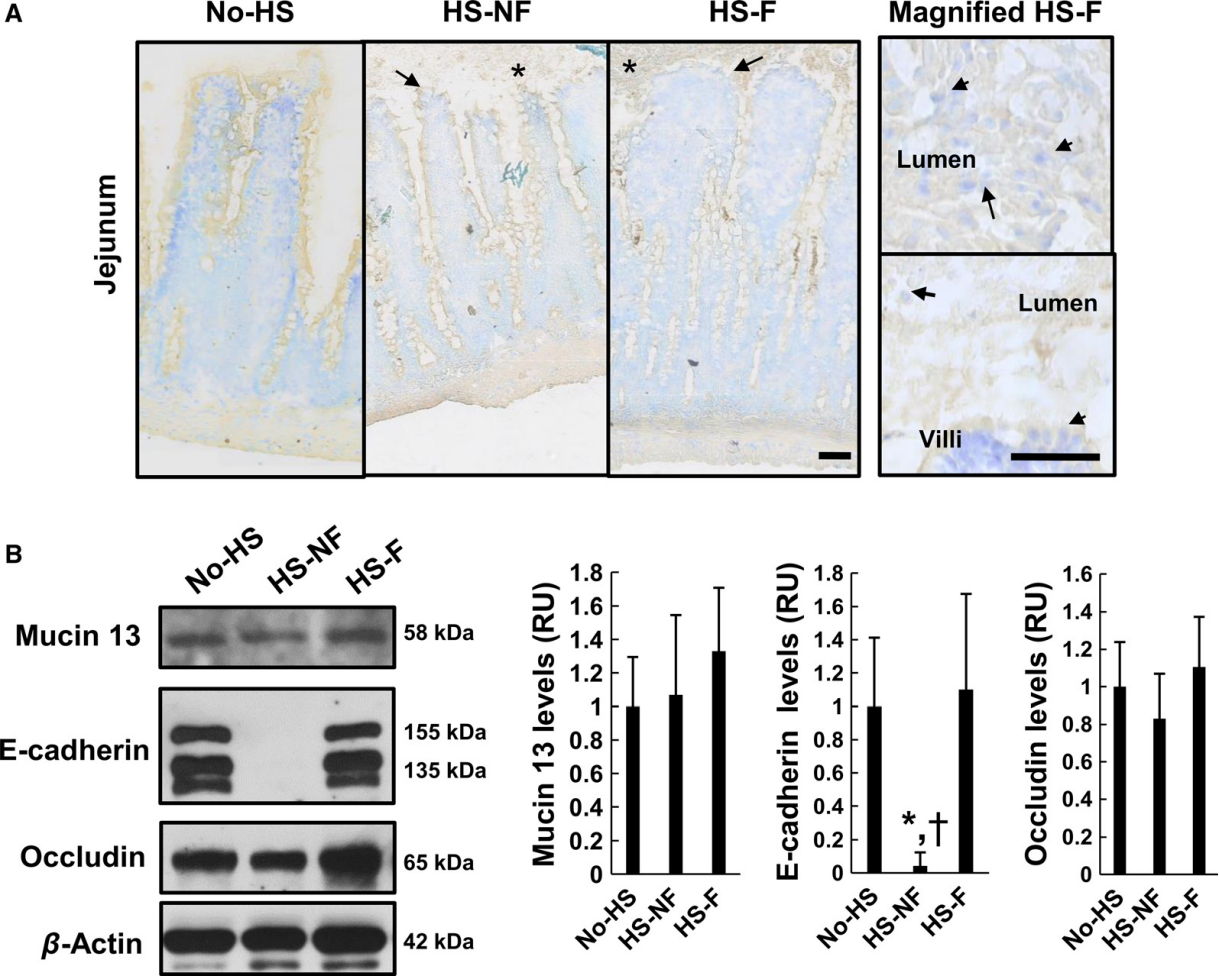
Mucosal barrier properties with and without luminal contents. (A) Mucin 13 (brown) labeling in the lumen (black arrows) on the intestine villi. Toluidine blue counterstain shows cell nuclei. (B) Immunblot of mucin 13, E-cadherin isoforms, and occludin (antibodies against intracellular protein domains). *P < 0.05 compared to No-HS and ^†^*P* < 0.05 compared to HS-F by ANOVA followed by Tukey post hoc test. N = 6 rats/group. Data is standardized to β-actin intensity and normalized by the No-HS levels. Bar graphs show mean ± SD. Scale bar corresponds to 30 μm.

Histological sections of the villi revealed shedding of epithelial bound mucin 13 into the lumen of the intestine after HS in both groups, though more pronounced in the HS-F group due to the lack of preexisting luminal contents (Fig. 2A). Although the distribution of mucin 13 changed after HS, the levels as determined by immunoblot did not (Fig. 2B). The E-cadherin bands of the intestinal epithelial cells were reduced in the HS-NF animals only, and the tight junction protein occludin band did not change (Fig. 2B).

### Transport of serine proteases from the gut

**Figure 3:**
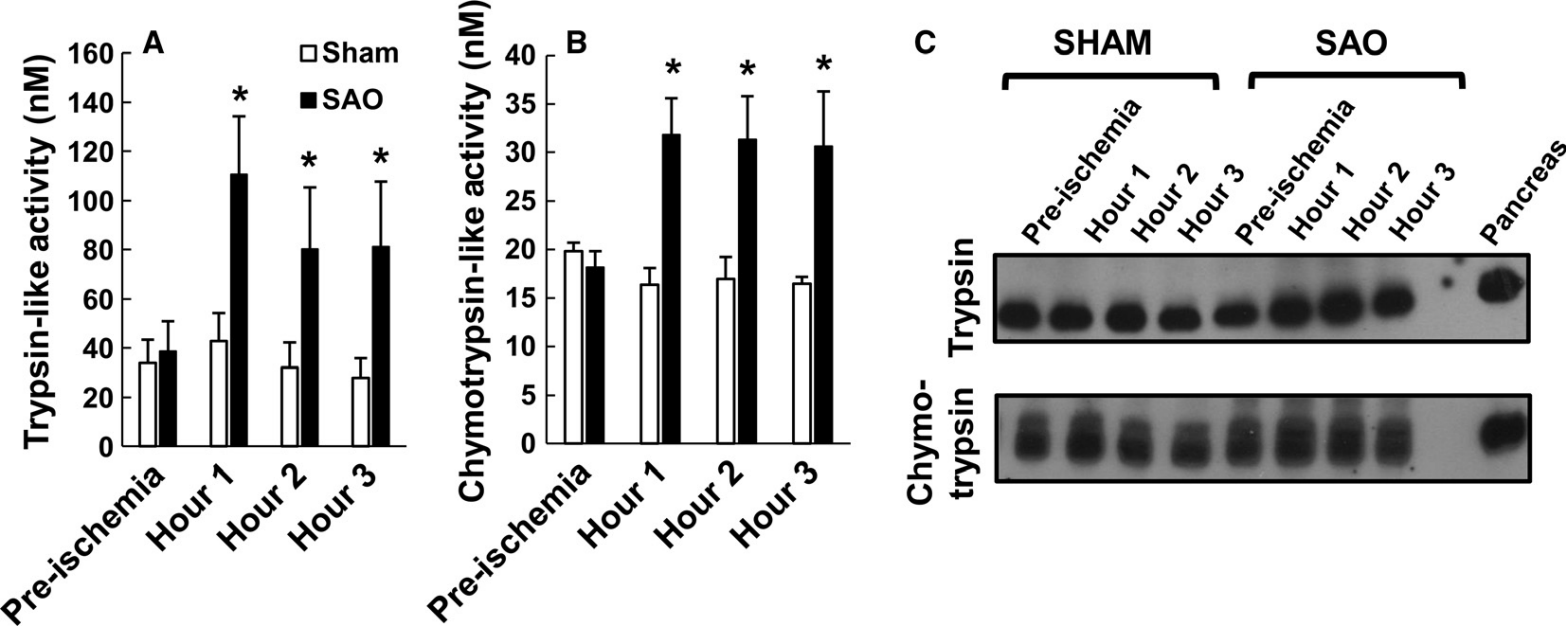
Protease activity in the lymph fluid after splanchnic artery occlusion (SAO) shock. Serine protease activity in the mesenteric lymph fluid of (A) trypsin and (B) chymotrypsin by cleavage of charge changing substrates. (C) Immunoblots of trypsin and chymotrypsin levels in the lymph using monoclonal antibodies specific to pancreatic trypsin and chymotrypsin. *P < 0.05 by *t*-test for each time point. Bar graphs show mean ± SD.

We collected mesenteric lymph fluid to study pancreatic enzyme activity and transport from the gut in animals with innate luminal contents after intestinal ischemia. Before intestinal ischemia, trypsin and chymotrypsin activities were detected in the nanomolar range, but after that the activities doubled and remained elevated throughout the reperfusion period (Fig. 3A and B). To confirm the presence of pancreatic trypsin and chymotrypsin in the lymph, monoclonal antibodies against rat pancreatic trypsin or chymotrypsin were used to successfully detect these proteins in the lymph fluid, although their levels did not change after intestinal ischemia (Fig. 3C).

**Figure 4:**
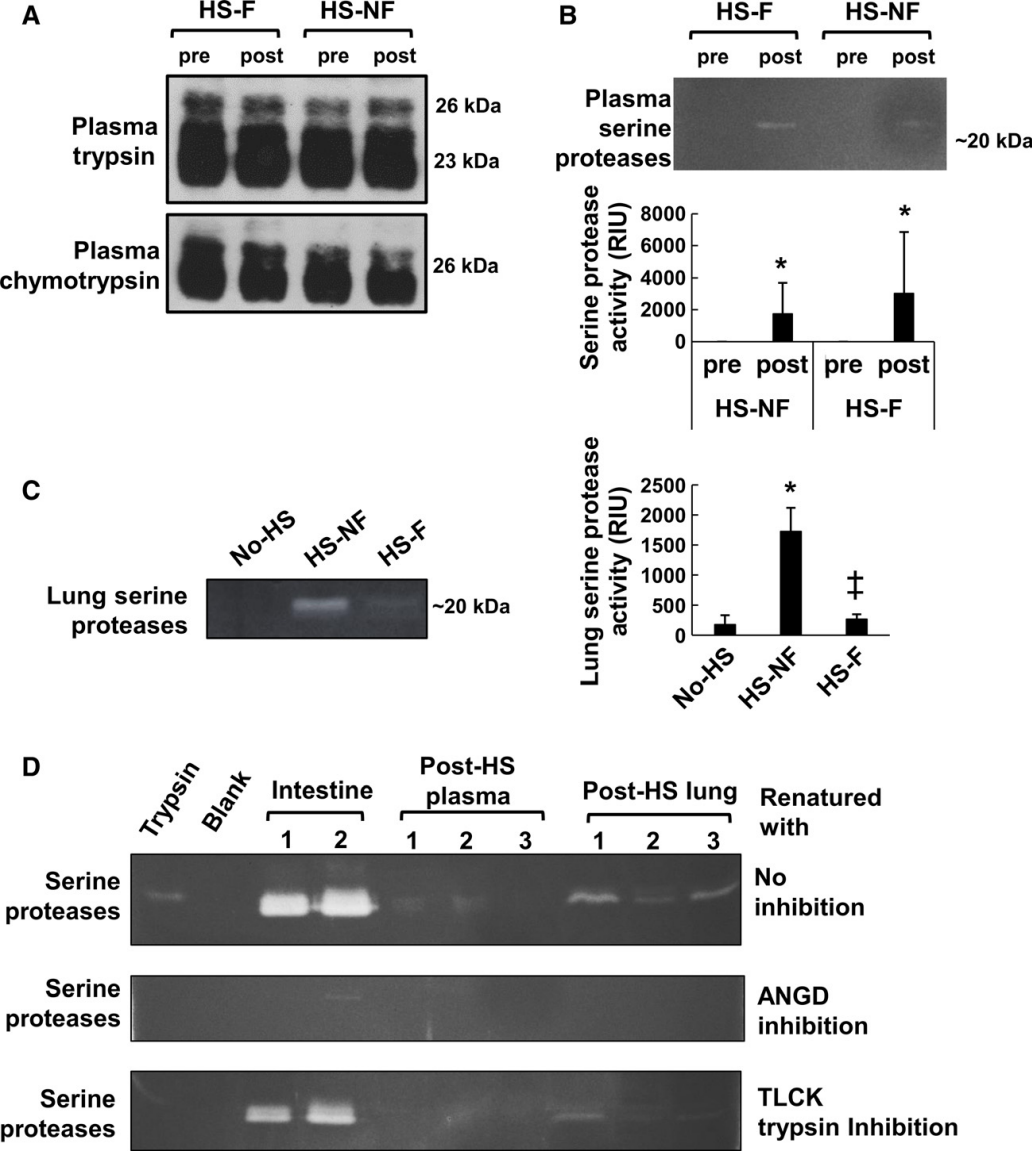
Serine proteases in the plasma and in the lung. (A) Pancreatic trypsin and chymotrypsin (were present in pre- and post-HS plasma samples at equal protein levels. (B) Activity bands corresponding to the molecular weight of serine proteases formed by gelatin gel zymography of plasma samples after HS (bar graph, mean±SD). *P < 0.05 by Mann–Whitney paired *t*-test compared to preplasma (undetectable in all preplasma samples). (C) Serine protease activity bands were detected only in the lung homogenates of those intestines that were not flushed. *P < 0.05 compared to No-HS and ^‡^*P* < 0.05 compared to HS-NF by ANOVA followed by Tukey post hoc analysis. N = 6 rats/group. (D) Gel zymography of post-HS lung homogenate samples showing low-molecular-weight bands forming around 20 kDa in lung homogenate (20 μg protein/lane), plasma (0.5 μl/lane), and intestine homogenate (2 μg protein/lane). Renaturating and developing with ANGD eliminated all protease bands. Inhibition with the trypsin-specific inhibitors reduced or eliminated the intensity in these bands.

Likewise, both trypsin and chymotrypsin were detected at equal levels in pre- and post-HS plasma in HS; flushing the intestine did not affect their levels (Fig. 4A). Low-molecular-weight bands (∼20 kDa) were present in gelatin gel zymography in post-HS plasma samples only in the HS-F and the HS-NF groups (Fig. 4B). As the lung is the first organ that mesenteric lymph fluid encounters upon mixing with venous return blood, we checked for low molecular weight band formation in the lung homogenates and found elevated activity in the HS-NF animals only (Fig. 4C). To confirm that these bands were serine proteases, we renatured gels with a broad spectrum serine protease inhibitor ANGD or trypsin-specific inhibitor (TLCK) and found a substantial decrease in the serine protease band intensities in the gels from plasma, lung, and intestine samples (Fig. 4D).

### MMP-9 activity and levels after HS in HS-F and HS-NF animals

**Figure 5:**
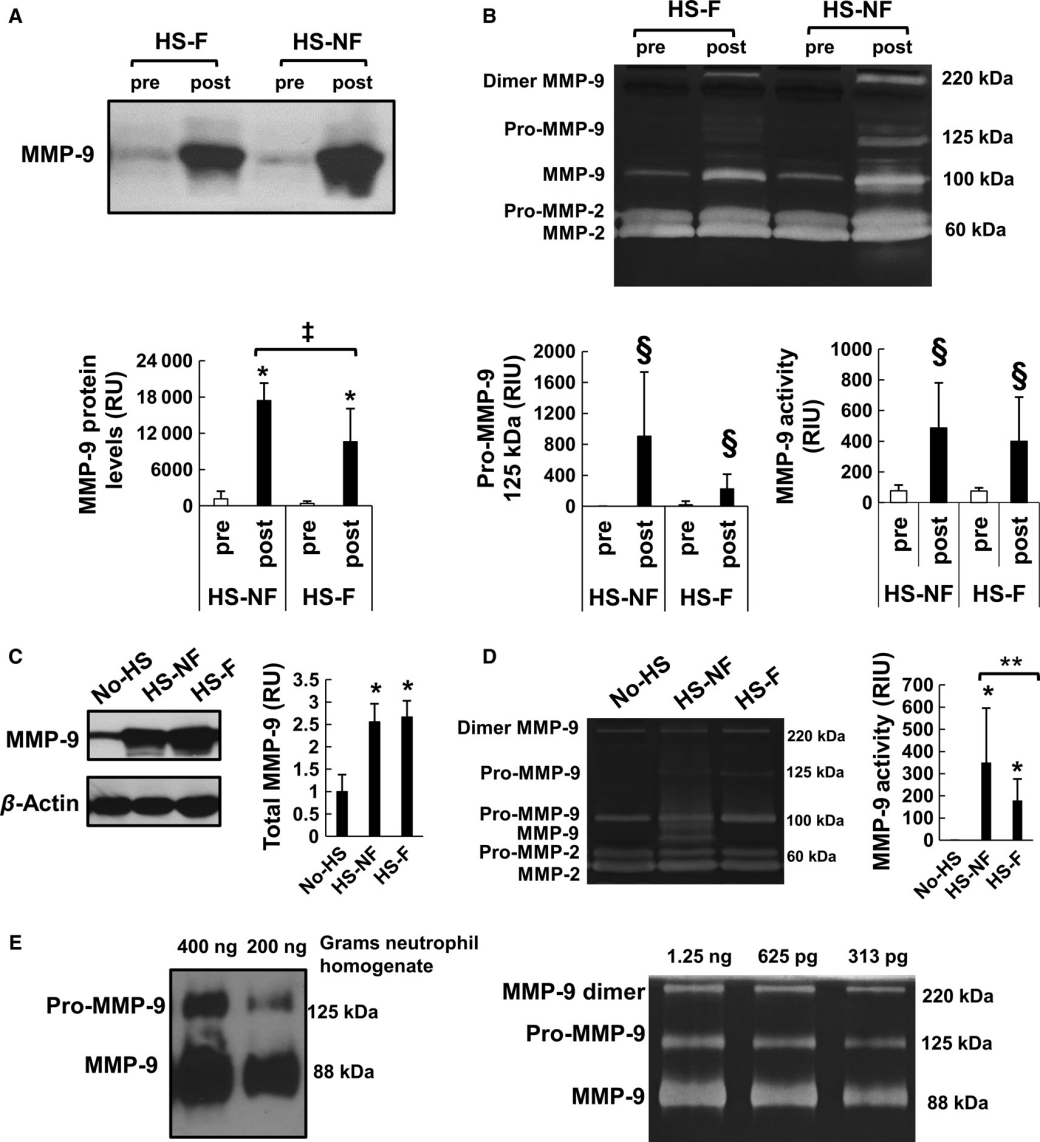
MMP-9 activity in plasma and lung. (A) MMP-9 pro-form and activity levels in plasma measured by gelatin gel zymography increased after hemorrhagic shock (HS) regardless of whether the intestine was flushed or nonflushed. *P < 0.005 by paired *t*-test between pre- and postsamples. ^‡^*P* < 0.05 by *t*-test between post-HS-F and post-HS-NF samples. (B) MMP-9 band corresponding to the sum of the MMP-9 dimer, pro- and active forms of MMP-9 after shock measured by immunoblot. ^§^*P* < 0.05 by Mann–Whitney test. (C) Protein levels of MMP-9 in the lung also increase after HS regardless of intestinal flush. (D) The activity bands corresponding to MMP in the lung measured by gelatin gel zymography. Bar graphs show mean ± SD. N = 6 rats/group. ^§^*P* < 0.025 by Mann–Whitney test. **P < 0.025 by Mann–Whitney test. (E) Corresponding activity levels detected by immunoblot showing the 125 kDa band corresponding to MMP-9 (samples were not reduced). Gelatinase activity by zymography of isolated neutrophils depicts three distinct bands for MMP-9, pro-MMP-9, and MMP-9 dimer at different dilutions (left).

Previous studies have shown that MMP-9 is elevated in the plasma and lungs after HS (Altshuler et al. 2012), and we chose to investigate whether the activation differed if luminal contents were present as active trypsin in the systemic circulation can directly convert pro-MMPs into their active forms (Duncan et al. 1998). The MMP-9 total protein levels (samples reduced by β-mercaptoethanol) in the plasma increased in both HS-F and HS-NF animals, but the HS-F animals were significantly lower compared to the HS-NF animals (Fig. 5A). After HS, the protease activity of both active MMP-9 and pro-MMP-9 (the pro-forms of MMP are activated by the denaturation/renaturation in gel zymography; 125 kDa derived from neutrophils [Olson et al. 2000]) increased after shock in the plasma in the HS-F and HS-NF animals (Fig. 5B). The MMP-9 dimers also increased after HS in the plasma, but there was no difference between the HS-NF and HS-F groups (not shown). There were no changes in the density of MMP-2 or pro-MMP-2 (not shown). The MMP-9 levels in the lung were elevated after HS in the lung homogenate as detected by immunoblot (Fig. 5C). Neutrophil-derived pro-MMP-9 (125 kDa) activity determined by gel zymography was elevated in both groups, and MMP-9 activity was also elevated but the HS-NF was significantly elevated compared to the HS-F group (Fig. 5D), possibly reflecting activation by active serine proteases in the lung (Fig. 4C). There were no changes in the levels of MMP-9 dimers that formed. We confirmed that these molecular weights are equivalent to the activity bands derived from neutrophils (Fig. 5E). There were no changes in MMP-9 or MMP-2 activity in the lymph fluid as determined by gelatin gel zymography between SAO and Sham animals throughout each time course (not shown).

### Lung injury in HS-F and HS-NF animals

**Figure 6:**
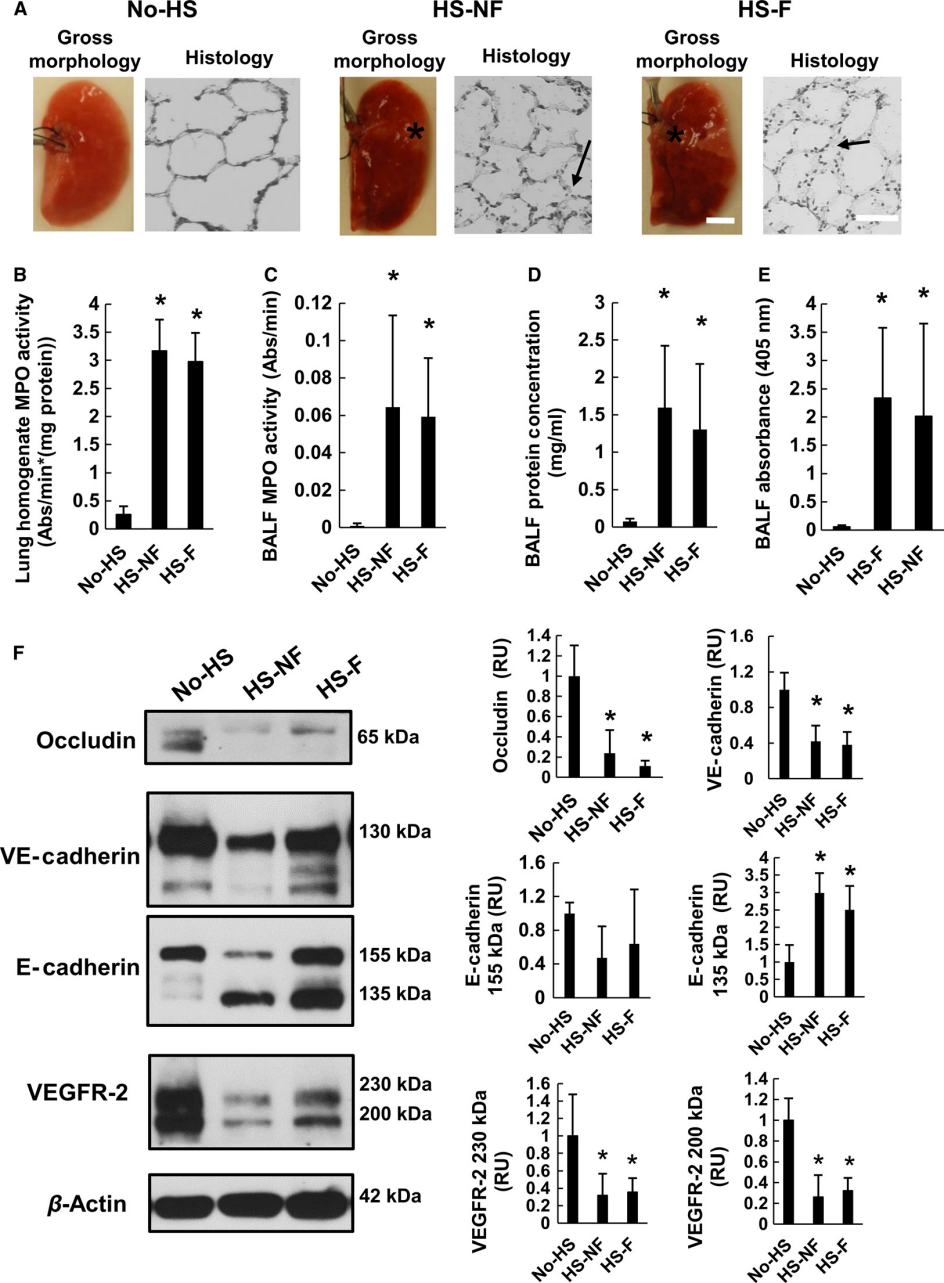
Markers for lung injury after hemorrhagic shock (HS). (A) Lung damage in form of microhemorrhages (see *) as observed by gross morphology. Histological sections of the most damaged lungs had visible swelling of the alveoli (arrows) in corresponding sample images magnified on the right. No-HS control exhibited no bleeding. (B) Lung MPO activity. (C) BALF protein concentration. (D) BALF MPO activity. (E) BALF absorbance were elevated after shock regardless of whether or not the intestine was flushed. (F) VE-cadherin, E-cadherin, occludin, and VEGFR-2 main isoforms in lung homogenates detected by immunoblot. They decrease following HS. Band densities are standardized to β-actin bands and normalized by No-HS levels. *P < 0.05 by ANOVA followed by Tukey post hoc. Bar graphs show mean ± SD. N = 6 rats/group.

Lung injury scores were 2.8 ± 1.1 and 2.9 ± 1.3, for the HS-F and HS-NF rats, respectively. Only one animal of the six without a flushed intestine had a low lung injury. Histology of the sections indicates swelling of the alveoli in both HS groups (Fig. 6A). The neutrophil accumulation as determined by MPO activity was elevated in both HS-F and HS-NF groups (Fig. 6B) and the BALF MPO was also elevated (Fig. 6C). The protein levels in the BALF were elevated after HS (Fig. 6D) and the absorbance due to free hemoglobin from red cell lysis, likely occurring in the circulation prior to extravasation, was increased (Fig. 6E).

The lung junctional proteins occludin, VE-cadherin, E-cadherin were degraded after HS regardless of whether the intestine was flushed (Fig. 6F). The endothelial survival protein VEGFR-2 also decreased after HS in both groups (Fig. 6F).

## Discussion

### Summary

**Figure 7:**
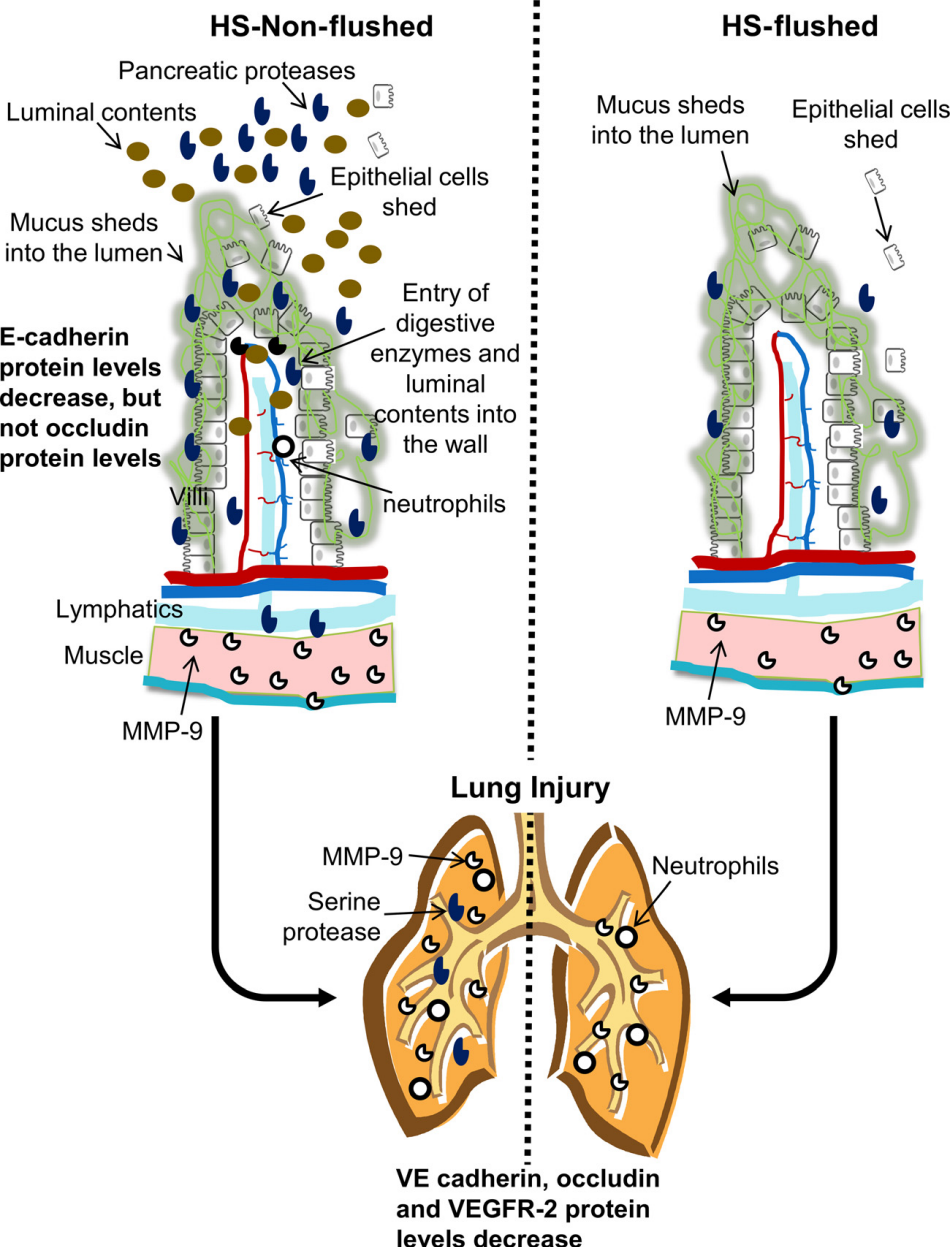
Summary. During hemorrhagic shock, intestinal epithelial cells shed into the lumen and the mucus breaks apart. If the intestine is not flushed, this allows digestive enzymes to penetrate into the wall and attract neutrophils. Digestive enzymes can transport from the intestine to peripheral organs through the lymph or portal venous fluid and accumulate in organs, such as the lung. In the absence of luminal contents, there is minimal destruction of the endothelial basement membranes reducing intestinal lesion formations as no luminal contents enter into the villi.

In HS, intestinal injury at the macroscopic and molecular levels is more severe if luminal contents are present prior to the onset of HS (HS-NF group). At lesion sites, red cell escape and neutrophil accumulation positively correlate with proteolytic activity in intestinal homogenates of the HS-NF animals (Fig. 7). However, the presence of luminal contents in the jejunum and ileum during HS affects neither the levels of serine proteases in the plasma, nor lung injury as measured by neutrophil infiltration and protein leakage. The protease activity increases in the mesenteric lymph fluid after intestinal ischemia, and the level of trypsin activity is increased in the lung in animals with a nonflushed intestine. Lung endothelial and epithelial proteins were degraded to a similar extent without and with the intestinal flush even though HS-NF animals had greater lung serine protease and MMP-9 activity and greater plasma total MMP-9 levels. The MAP of animals did not correlate with the degree of intestinal injury in the animals, indicating that at an early stage of fluid resuscitation degrading mechanisms may not necessarily be reflected in the blood pressure.

### Intestinal injury severity increases if luminal contents are present

After HS, the intestinal villi structure remains intact (Fig. 2A) compared to its state in SAO models (Ikeda et al. 1998; Mitsuoka and Schmid-Schönbein 2000; Grossmann et al. 2002; Chang et al. 2012b). The macroscopic hemorrhagic lesions in which red cells enter the intestinal wall and the lumen are a common feature after shock (Chiu et al. 1970a; Manohar and Tyagi 1973; Haglund et al. 1975), but they are not formed if the intestine is flushed (Fig. 1D). Furthermore, the strong correlation between intestinal wall hemorrhage and proteolytic activity of the intestinal homogenate (Fig. 1B and D) suggests that the digestive proteases play a role in the formation of these vascular lesions. Mucosal barrier damage is less severe if animals are fed an elemental diet restricting the pancreatic enzymes as opposed to normal nutrition (Bounous et al. 1967), which supports the idea that nutrients are essential for mucosal barrier protection (Chiu et al. 1970b; McArdle et al. 1972; Kozar et al. 2002), but proteases can be detrimental if this barrier breaks open during ischemia.

Our group has shown previously that intestinal ischemia can result in transport of pancreatic proteases into the lamina propria (Chang et al. 2012a,b). The requirement for these enzymes to enter the intestinal wall (and subsequently for red blood cells to enter the intestinal lumen) is that the mucosal barrier must fail. Following HS, regardless of the state of flushing, epithelial cells were present in the lumen (Fig. 2A) similar to previous reports, which indicate epithelial shedding into the lumen after intestinal ischemia (Bounous et al. 1967; Vakonyi et al. 1977; Robinson et al. 1981; Ikeda et al. 1998). This evidence suggests that even though the villi appear intact, openings in the barrier occur as epithelial cells are shed together with their attached mucin, providing pathways for pancreatic enzymes and luminal contents to enter the intestinal wall.

As pancreatic proteases may promote further mucosal barrier failure by cleaving interepithelial adhesion proteins, we investigated epithelial junctional protein levels. The presence of luminal content did not affect the levels of the tight junctional protein occludin (Fig. 2B). However, the adheren junctional protein E-cadherin was degraded predominantly in the nonflushed animals (Fig. 2B), which is consistent with previous evidence that E-cadherin degrades during intestinal ischemia, possibly by the digestive enzymes (Chang et al. 2012a,b).

Pancreatic proteases are not the only proteases entering or activated in the intestinal wall during HS. When whole intestines (wall plus lumen) of No-HS and HS-NF animal intestines were homogenized with their luminal contents the majority of the caseinolytic activity is derived from luminal pancreatic enzymes (Penn et al. 2007; Penn and Schmid-Schönbein 2008), which is expected to be similar between the two groups. However, the HS-NF animals exhibited higher levels of caseinolytic activity suggesting there may exist an additional source of proteases present in the HS-NF animals (Fig. 1B). As MPO activity also increased in intestinal homogenates in the HS-NF animals (Fig. 1F) indicating neutrophil accumulation, neutrophil-derived elastase or MMPs could be an additional source of proteolytic activity. Alternatively, pancreatic proteases may convert pro-MMPs (or other proteases) into active MMPs (Duncan et al. 1998; Rosario et al. 2004) yielding a net increase in protease activity.

Studies using enteral treatment with the serine protease and lipase inhibitor ANGD in the small intestine have shown reduced microhemorrhages in the intestine and improved outcomes in experimental shock (Mitsuoka and Schmid-Schönbein 2000; Doucet et al. 2004; Shi et al. 2004; DeLano et al. 2013), which supports the hypothesis that digestive enzymes or their products are responsible for the lesions. If the pancreatic enzymes enter the wall but are inactive, they would be unable to trigger a proteolytic activation cascade of MMPs (Fig. 5B and D) or degrade epithelial proteins (Fig. 2B). It should be noted that ANGD is solubilized in a glucose solution in those studies, and glucose itself serves to attenuate disintegration of the epithelial barrier during intestinal ischemia (Chiu et al. 1970a,b; Mirkovitch et al. 1975; Kozar et al. 2002). However, failure of the epithelial barrier alone does not appear to be sufficient to cause microhemorrhages in the intestine as there were no hemorrhages in HS-F animals despite the lack of a source of metabolic energy in the lumen of the intestine.

Development of microhemorrhages also requires that the endothelial cells lining the blood vessels in the intestine must fail. This may occur by a number of possible mechanisms. Pancreatic proteases, activated MMPs, or neutrophil elastase (Fig. 1C) may digest the basement membrane and endothelial cell adhesion proteins allowing red cells to escape into the interstitial space (Allport et al. 2002; Hu et al. 2005). The proteases may cleave extracellular matrix or dietary proteins into bioactive peptides that increase vessel leakage, or cleave fatty acid binding proteins allowing unbound free fatty acids to act as detergents destroying endothelial cells (Penn and Schmid-Schönbein 2008; Davis 2010; Qin et al. 2012). Unbound free fatty acids may also lyse erythrocytes (Lujan and Bronia 1994), which could allow free hemoglobin to escape into the lumen (potentially bypassing the need for extracellular matrix or basement membrane breakdown). Bioactive peptides or free fatty acids may also be proinflammatory (Badwey et al. 1984; Ferrante et al. 1994; Penn et al. 2007, 2012) contributing to the neutrophil accumulation.

Although bacteria in the gut can be a potential injury mechanism for the intestine (Yale 1969; Musemeche et al. 1986; Deitch et al. 1991), they tend to be distributed throughout the gut with higher levels in the ileum regions compared to the jejunum (Salanitro et al. 1978; Fryer et al. 1996). However, the intestinal injury was greatest at locations where food boluses were present and where digestive enzymes are most concentrated.

### Serine protease transport occurs already before shock, but activity increases after HS

The major translocation routes for luminal content from the intestine into the circulation can be directly via the portal venous circulation, the mesenteric lymph, or the peritoneal space and the lymphatics surrounding the peritoneum (Deitch 2010; Altshuler et al. 2012). Interestingly, trypsin and chymotrypsin were present in the mesenteric lymph even before the onset of shock, but the trypsin-like and chymotrypsin-like activities were low. After shock, the degree of protease activity in the lymph was on the order of nmol/L, which is in the range of previous measurements in post-HS plasma (Altshuler et al. 2012). The elevation of protease activity in the lymph but the lack of change in the protease levels suggests that trypsin and chymotrypsin were being activated, inhibitors were deactivated, or that the activity was from other enzymes with similar specificity. As zymogen trypsin and chymotrypsin secreted into the intestine are rapidly converted to their active forms, if the change after shock is due to activation, then it is likely that the zymogen enzymes present before shock originated directly from the pancreas rather than from the intestine, though the active enzymes responsible for their conversion during shock may originate from either organ (note that unlike MMPs, pancreatic serine zymogen proteases are not activated by gel zymography). Past proteomic studies of postshock mesenteric lymph have not reported trypsin or chymotrypsin in the lymph fluid, possibly because these enzymes are commonly used to digest samples prior to mass spectrum analysis (Fang et al. 2010; Zurawel et al. 2010). The significance of elevated protease activity in the lymph could have implications on lymphatic endothelial cell extracellular damage and degradation of proteins in the lymph fluid.

The pancreatic proteases were also present in all plasma samples before the onset of HS, but enzyme activity by zymography was undetectable (Fig. 4A and B). As electrophoresis would be expected to separate the enzymes from any plasma protease inhibitors, this supports the idea that these are zymogen proteases from the pancreas. However, the protease activity in gel zymography at low-molecular-weight increased in post-HS plasma, regardless of the luminal contents (Fig. 4B), similar to previous findings (Altshuler et al. 2012). Possibly they are being activated during shock by pancreatic ischemia or in the lymph or plasma by small quantities of active enzymes from the intestine. Inhibition of these enzymes with ANGD and TLCK eliminated the bands, suggesting that they are pancreatic enzymes (Fig. 4D) instead of MMPs or plasmin (Frederiks and Mook 2004).

Unlike plasma and lymph, which had no significant difference in serine protease activity between flushed and nonflushed groups following HS, lung serine protease activity was higher in the nonflushed case than the flushed case (Fig. 4C). We have seen previously that not only trypsin activity, but trypsin protein levels increase in the lung after HS (Altshuler et al. 2012). The lack of increase in the flushed group suggests that the enzymes are intestinal in origin. The lung is the first capillary bed encountered upon discharge of lymphatic fluid via the thoracic duct into the venous circulation. Therefore, active proteases from the lymph may become entrapped in the lung upon mixing with the venous blood rather than circulating systemically because plasma proteins can accumulate in the lung after shock (Staub 1981).

### MMP-9 accumulation in tissues after HS

The intestine's MMP-9 levels increased only in the HS-NF animals (Fig. 1C) and may be due to increased neutrophil accumulation in the wall, an observation that is supported by the increased MPO activity (Fig. 1F). Aside from the intestine, the MMP-9 protein levels in the plasma were elevated in groups with or without a flushed small intestine suggesting that MMP-9 secretion into the plasma did not depend on the luminal contents (Fig. 5A). MMP-9 levels and activity also increased in the lung during hypotension in both the HS-F and HS-NF animals (Fig. 5C and D). The active form of MMP-9 in the lung was nearly doubled in the HS-NF group compared to the HS-F group (Fig. 5D). The increase in MMP-9 activity may be due to activation by serine proteases transported into the lung (Fig. 4C) (Duncan et al. 1998).

### Lung inflammation

Although there was more MMP-9 and serine protease activity in the lungs in the HS-NF animals, the BALF protein levels and MPO activity did not differ between groups after HS (Fig. 6C–E). Enteral protease inhibition with ANGD has been shown to attenuate MPO infiltration into the lungs in a similar model of HS (Altshuler et al. 2012), which, combined with our findings, suggests that the protease inhibition is acting at sites distant from the intestinal lumen. Paradoxically, these same pancreatic protease inhibitors are not effective at attenuating organ injury if administered systemically instead of enterally (Deitch et al. 2003; Shi et al. 2004). This suggests that neutrophil activators may be arising from multiple locations in the body during HS, and that if either the intestine or the alternate source is ignored, neutrophils may become activated resulting in lung injury. Given that the pancreas contains proteases and during ischemia could generate many of the same mediators generated by the intestine, the pancreas may be an alternative source of inflammatory mediators (Kistler et al. 2000). This hypothesis is supported by reduced lung damage in studies that ligate the mesenteric lymph vessel as a carrier of mediators from both intestine and pancreas (Deitch 2010). Intravenous ANGD is an approved treatment for pancreatitis in Japan, supporting the idea that enteral ANGD, if absorbed into the circulation or leaked into the peritoneal space, could reduce damage and mediator release in the pancreas.

### Protein degradation in the lung

VE-cadherin, an adhesion protein expressed on lung endothelial cells (Shasby 2007), occludin, a tight junction protein present on both endothelial and epithelial cells in the lung, and both isoforms of the endothelial growth receptor VEGFR-2 were reduced after HS regardless of flushing the luminal contents (Fig. 6F). E-cadherin, another endothelial adhesion protein also (nonsignificantly) decreased after shock, and there was a significant increase in a smaller molecular weight form of the protein suggesting cleavage by a protease (Fig. 6F). Neutrophils accumulate in the lung during shock (Fig. 5C and D) and could therefore contribute to the loss and/or cleavage of these proteins, as neutrophil elastase and MMPs have been documented to contribute to the destruction of cadherins (Carden et al. 1998; Navaratna et al. 2007), possibly as part of neutrophil transmigration. The loss of any or all of the proteins tested here could contribute to increased lung permeability. The reduction of the endothelial survival receptor VEGFR-2 could impair vascular repair following the ischemic injury (Holmes et al. 2007). However, as all of these proteins were degraded regardless of the presence of luminal content, the mechanism is likely independent of the proteases present in the lumen of the small intestine and more dependent on MMPs activated in the lung during ischemia. Though damage was independent of protease activity at the end point for this study (3 h postreperfusion), it is possible that the accumulated protease activities in the lung could cause increased damage at later time points or to a greater extent in other animal models or humans. Additionally, other proteins in the lung may also be degraded and could be affected by the increase in proteolytic activity from the luminal contents present in the intestine.

### Limitations

Although flushing the intestinal luminal contents is a useful experimental technique to determine which indices of organ damage are influenced by intestinal luminal factors during shock, new technologies may be need to be introduced to flush the luminal contents from the intestine in critical care patients. Other limitations of this model include that while the jejunum and ileum regions were flushed to match the regions previously given enteral protease inhibitors, the duodenum, cecum, and large intestine contained luminal contents (though we have observed protease activity is greatly decreased by the time the content reaches the terminal ileum, cecum, and large intestine and is therefore less of a concern; unpublished observations). Also, several of the same mediators that could originate in an intestine in the presence of its luminal content may also be generated in an ischemic pancreas (Kistler et al. 2000) and would be unaffected by flushing the small intestine's lumen. In the current experiments we chose not to control the food intake of any of the animals; consequently the animals had variable amounts of food in different regions of the intestine, which may explain some of the variability in the measurements.

## Conclusions

Comparison of the early outcomes of HS with or without the luminal contents in the intestine reveals an increased intestinal damage in the presence of luminal contents. This observation may be of use to assess the potential severity of intestinal damage in trauma patients. While the luminal contents increased the intestinal lesion formation and protein degradation after HS, removing luminal content reduced damage in the intestine but did not reduce all molecular indicators for lung injury, suggesting that mechanisms may be involved in peripheral organ damage other than just those derived from the luminal contents in the small intestine. Accumulation of active pancreatic proteases in intestinal lymph fluid and protease activity in the lungs after shock suggests a potential mechanism which involves activation of MMP-9 in the lungs in both flushed and nonflushed animals. The progression of organ failure depends upon degrading proteases, and future studies need to investigate methods to prevent the escape of the luminal contents past the epithelial barrier as well as alternative options to reduce lung injury following HS.
